# Comprehensive identification, characterization and expression analyses of the class III POD gene family in water lily (*Nymphaea colorata*)

**DOI:** 10.3389/fpls.2024.1524657

**Published:** 2025-01-20

**Authors:** Wasi Ullah Khan, Latif Ullah Khan, Noor Muhammad Khan, Ji Zhang, Wang Wenquan, Fei Chen

**Affiliations:** ^1^ National Key Laboratory for Tropical Crop Breeding, College of Breeding and Multiplication, Sanya Institute of Breeding and Multiplication, Hainan University, Sanya, China; ^2^ College of Tropical Agriculture and Forestry, Hainan University, Danzhou, China

**Keywords:** plant peroxidases, water lily, expression pattern, phylogenetic analysis, stresses

## Abstract

Class III peroxidases are plant-specific glycoproteins and widely distributed among plant species, that play a crucial role in plant resistance to different stresses, such as salt, heat, cold and metal toxicity. The present study is the first comprehensive and systematic report to characterize the *NcPOD* gene family in water lily (*Nymphaea colorata*). In this study, 94 *NcPOD* genes in water lily were identified, each possessing a conserved *POD* domain, which are dispersed unevenly across the genome. Through comparative maximum-likelihood phylogenetic analysis, these genes were categorized into 10 groups, along with two other species, *Arabidopsis thaliana* and *Nymphaea thermarum*. Notably, the largest group, group-c, comprised 32 distinct types of NcPOD proteins. These genes exhibited uneven distribution on 11 of the 14 chromosomes of water lily. Exon-intron and motif analyses exhibited the structural and functional diversity among the sub-groups. The Examination of duplication patterns suggests that tandem duplication has contributed to the expansion of *NcPOD* genes. The analysis of promoter cis-acting elements indicated the presence of regulatory elements associated with various responses such as ABA, MeJA, light responsiveness, anaerobic conditions, and drought inducibility. Finally, the RT-qPCR based expression and enzymes activity of ten *NcPOD* genes depicted the dynamically differential response to NaCl, heat, cold, and heavy metals (CuSO_4_ and CdCl_2_) stresses. These findings provide valuable insights for future exploration of *NcPOD* functions in water lily growth and stress tolerance, laying a foundation for further comparative genomics and functional studies of this important class of antioxidant genes.

## Graphical Abstract

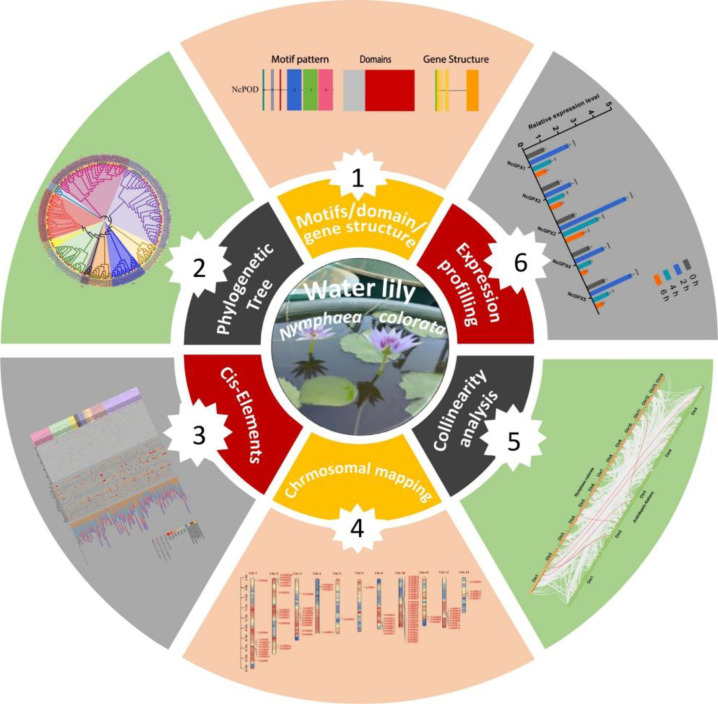

## 1 Introduction

Peroxidases, often referred to as PODs, represent a diverse group of enzymes responsible for catalyzing the oxidation of numerous substrates by utilizing hydrogen peroxide (H_2_O_2_) as an oxidizing mediator. These enzymes are found in all living organisms and can be categorized into different families based on their structural and catalytic characteristics (Pandey et al., 2017). Among them, one particular family, class III peroxidases (EC 1.11.1.7), is unique to the plant kingdom. Enormous varieties of POD isozymes encoded by a multigene family has been reported within higher plants (Passardi et al., 2004b; Kidwai et al., 2020). However, the abbreviation used in the papers differs as POD or PRX. Though, in this study, POD was used to distinguish it from another group of peroxidases.

Over the past few decades, extensive research has been conducted to unravel the functions of *POD* genes across various plant species. These genes have been confirmed to play a vital role in a wide range of physiological and developmental processes. Their functions include cell elongation, the creation of cross-links within cell wall components, the formation of lignin and suberin, the scavenging of reactive oxygen species (ROS), wound healing, phytoalexin synthesis, and defence responses against both abiotic and biotic stresses (Hiraga et al., 2001; Gabaldón et al., 2005; Passardi et al., 2005; Daudi et al., 2012; Qiang et al., 2023). Additionally, *PODs* have the capability to catalyze the phytohormone auxin and produce hydroxyl radicals and H_2_O_2_, which play intricate roles in defence signalling (Gaspar et al., 1985; Joo et al., 2001). Numerous functional studies have previously highlighted the role of *PODs* in enhancing crop plants’ tolerance to various environmental stresses (Obaid et al., 2024). For instance, when *Arabidopsis* peroxidase 64 (*AtPrx64*) was overexpressed in transgenic tobacco plants, they exhibited enhanced tolerance to aluminium stress (Wu et al., 2017). Similarly, the cold-inducible gene *RCI3* (*AtPrx3*) encoded a *POD* in *A. thaliana*, providing increased tolerance to salt and drought stresses (Llorente et al., 2002). The germination rate was improved by overexpressing the peroxidase genes extracted from *Catharanthus roseus* (*CrPrx* & *CrPrx1*) against cold, salt and dehydration stresses (Kumar et al., 2012). In tomatoes, reduced susceptibility to bacterial fleck was achieved through the down-regulation of the *Ep5C* gene, encoding a *POD* (Coego et al., 2005). Knock-out lines of pepper lacking the extracellular gene *CaPO2* displayed increased vulnerability to bacterial pathogens, while overexpression of *CaPO2* conferred enhanced resistance (Choi et al., 2007). Furthermore, transgenic carrot plants overexpressing the rice cationic *POD* gene *OsPrx114* exhibited heightened resistance to fungal diseases (Wally and Punja, 2010). Some maize *POD* genes are under the regulation of hormones and pathogen elicitors (Mika et al., 2010). Similarly, the overexpression of *GsPRX9* in soybean composite seedlings led to improved tolerance to salt stress (Jin et al., 2019). These collective findings accentuate the positive contribution of class III plant peroxidases in responding to both biotic and abiotic stresses.

In various plant species, comprehensive whole-genome analyses have unveiled the members of the class III *POD* family, highlighting their essential roles in both defence responses and the regulation of plant growth and development. For example, tobacco (*Nicotiana tabacum*) comprised (210) (Cheng et al., 2022), *Arabidopsis* found 73 *PODs* (Tognolli et al., 2002; Duroux and Welinder, 2003; Valério et al., 2004), rice (*Oryza sativa*) contained 138 *PODs* (Passardi et al., 2004a), *Populus trichocarpa* had 93 *PODs* (Ren et al., 2014), *Medicago sativa* possessed 102 *PODs* (Behr et al., 2015), maize encompassed 119 *PODs* (Wang et al., 2015), and 94 *PODs* were identified in *Pyrus bretschneideri* (Cao et al., 2016).

Water lilies are aquatic plants of global horticultural and economic importance and among the earliest diverging angiosperms – Darwin’s abominable mystery –150 million years ago (Mya) (Zhang et al., 2020). The water lily (order Nymphaeales), comprises around 100 species of aquatic herbs, and plays a crucial role in evolutionary biology (Xiong et al., 2023; Khan et al., 2024). These plants are classified within the ornamental-rich genus Nymphaea (Borsch et al., 2007). Numerous Nymphaea species are globally recognized as important aquatic plants, known for their different colors, prolonged blooming periods, high stress tolerance, and adaptability (Chen et al., 2017). Their flowers are mostly used in tea making, as fresh cut flowers, and as well as in textile production (Khan et al., 2023). The water lily specie *Nymphaea colorata*, commonly known as blue pygmy or colorata, is a highly valued ornamental aquatic plant admired for its vibrant flower colours and attractive shapes. Its small size and nonviviparous nature make it ideal for small- to medium-sized water gardens. The plant feature leaves around 10 cm in diameter, which are green on the upper surface and have a bluish-violet color on the underside of the leaf. The flowers, are cup-shaped, and violet-blue, with a paler tone at the base of the petals and stamens (Zhang et al., 2020; Khan et al., 2024). They are also slightly fragrant, increasing their ornamental demand. These distinctive traits have contributed to the growing popularity of *N. colorata* in aquatic gardens worldwide. Furthermore, the species has been utilized in breeding programs across the globe. The first water lily (*N. colorata*) genome sequence was released in nature in 2020 (Zhang et al., 2020), though the *POD* gene family has not yet been identified in any species of water lily.

In the current study, we conducted a comprehensive bioinformatics analysis of the *POD* gene family of water lily and elucidated their roles in responding to various abiotic stressors, including NaCl, heat, cold, and heavy metals. We successfully identified a total of 94 genes within the water lily genome for the first time, subjecting them to thorough genome-wide analysis. This analysis encompassed the examination of physicochemical properties, phylogenetic relationships, motif composition, gene structure, promoter sequences, RT-qPCR, and enzymes accumulation in response to abiotic stresses. The conclusions of this study are expected to serve as a valuable resource for future research on water lily species and lay the base for the functional characterization of the *POD* gene family.

## 2 Materials and methods

### 2.1 Sequence data retrieval and identification of *NcPOD* genes

All *NcPODs* including genomic and coding sequences were obtained from the Phytozome database. The 73 *POD* genes of *Arabidopsis* (http://www.arabidopsis.org/index.jsp accessed on 15 March 2024) had their protein sequences obtained from the corresponding database (Lamesch et al., 2012). To identify *POD* genes in the water lily genome, the *Arabidopsis* POD sequences were used as queries. Additionally, an HMMER-based search was conducted to predict POD proteins in the water lily genome (Potter et al., 2018). Then, all the predicted *POD* sequences underwent further validation through BLAST searches using the Pfam (PF00141) (http://pfam.xfam.org/accessed on 19 March 2024), and conserved domain (CDD) databases (Finn et al., 2007; Marchler-Bauer et al., 2015). In last all, the *POD* genes obtained from HMMER and BLAST were scrutinized using SMART (http://smart.embl-heidelberg.de/ accessed on 20 March 2024) to confirm the presence of *POD* conserved domains (Letunic et al., 2012). Finally, any redundant proteins or proteins identified as ascorbate peroxidases were manually reviewed and excluded.

### 2.2 Examination of protein characteristics and in-silico subcellular localization

The ExPASy proteomics server (http://web.expasy.org/protparam/) was used to assess the physiochemical properties of NcPOD proteins. This analysis included parameters such as the number of amino acids, chromosome length, molecular weight (MW), isoelectric points (pI), and the grand average of hydropathicity (GRAVY) (Gasteiger et al., 2003). To predict the subcellular localization of POD proteins, the WoLF PSORT (https://wolfpsort.hgc.jp/ accessed on 25 March 2024) (Edgar, 2004) and the ProtComp 9.0 server (http://linux1.softberry.com/ accessed on 30 March 2024) was employed (Khan et al., 2023).

### 2.3 Analysis of conserved motifs and *NcPOD* domains

The MEME program with default settings (https://meme-suite.org/meme/db/motifs accessed on 5 April 2024) was used to identify conserved motifs within all protein sequences (Bailey et al., 2009). Subsequently, TBtools (V 1.068, https://github.com/CJ-Chen/TBtools/ accessed on 7 April 2024) was employed to visualize the phylogenetic tree, conserved motifs, and conserved domains of the *NcPOD* family genes (Chen et al., 2020).

### 2.4 Phylogenetic analysis of POD proteins

To create a phylogenetic tree, the POD protein sequences identified in *N. colorata*, *A. thaliana*, and *N. thermarum* were used. Alignment of the PODs was conducted employing the CLUSTALW method (Thompson et al., 1994). Subsequently, the phylogenetic tree for the POD family proteins was constructed using the maximum-likelihood model in MEGA-X (https://megasoftware.net/home accessed on 15 April 2024) (Kumar et al., 2016) with 1000 bootstrap replicates. Finally, the resulting phylogenetic tree was improved through the online tool iTOL (https://itol.embl.de/; accessed on 17 April 2024) (Letunic and Bork, 2021).

### 2.5 Mining cis-regulatory elements in *NcPODs*

The 2000 bp upstream regions was mined from the transcription start sites for all *NcPODs*, obtaining the data from the Phytozome database. These sequences were then subjected to a search for potential cis-acting regulatory elements through the PlantCARE tool (https://bioinformatics.psb.ugent.be/webtools/plantcare/html/ accessed on 27 April 2024) (Lescot et al., 2002). The results of this analysis were manually processed and visualized using the ‘Basic Biosequence View’ feature in TBtools v1.108 software (Chen et al., 2020).

### 2.6 Gene structure, chromosomal location and synteny analysis

The CDS sequences of *PODs* were obtained from the genomic structure information (GFF) of the genome and then visually examined their intron and exon structures utilizing the Gene Structure Display Server (GSDS; http://gsds.cbi.pku.edu.cn/index.php accessed on 29 April 2024) (Guo et al., 2007). To generate a schematic diagram, TBtools were employed. The chromosomal locations of *NcPOD* family genes in the *N. colorata* genome were obtained and visualized using the GFF3 annotation file with TBtools v.2.069 software (Chen et al., 2020). Genome assemblies of both *N. colorata* and *A. thaliana* were utilized to calculate synteny and collinearity and create a dual synteny plot using the MCScanS program within TBTools (V 1.068, https://github.com/CJ-Chen/TBtools/ accessed on 1 May 2024) (v1.0692).

### 2.7 Plant materials and abiotic stresses

To investigate the response of *N. colorata POD* antioxidant genes to various abiotic stresses, including salinity (250 mM), heat (42°C), cold (08°C), and heavy metals (Cu; CuSO_4_ 200 µM and Cd; 2.5 mM CdCl_2_), experiments were conducted (Khan et al., 2023). The water lily (*N. colorata*) plants were obtained from National Key Laboratory for Tropical Crop Breeding, College of breeding and multiplication, Sanya Institute of Breeding and Multiplication, Hainan University, Sanya, China. The mature *N. colorata* plants were grown in a water tub filled with tap water under an open atmosphere. Each treatment consisted of three separate biological replicates, with samples collected from at least five individual plants. Leaves from the plantlets were collected at 0, 2, 4, and 6 h for the heat, salt, cold, and heavy metal stress. After collection, all samples were immediately frozen in liquid nitrogen and stored at −80°C until total RNA extraction.

### 2.8 RNA extraction and real-time quantitative PCR expression analysis

RNA was extracted utilizing the RNAprep Pure Plant Kit (TIANGEN, Beijing, China), and the concentration of the samples was measured with a NanoDrop 2000 C spectrophotometer (Thermo Fisher Scientific, Waltham, MA, USA). Removal of genomic DNA was accomplished through DNase I treatment, followed by cDNA synthesis using the QuantiTect Reverse Transcription Kit (Qiagen, Shanghai, China). The RT-qPCR expression was performed on the Roche LightCycler 96 PCR system, following the recommended guidelines for the ChamQTM SYBR RT-qPCR Master Mix (Vazyme Biotech Co., Ltd., Sanya, China). Gene-specific primers (S1) for *NcPOD* and Nc-Actin were designed using the online NCBI Primer-BLASTProgram and their specificity was confirmed utilising the Oligo Calculator online tool (http://mcb.berkeley.edu/labs/krantz/tools/oligocalc.html accessed on 10 June 2024). Three biological replicates for each sample were employed for RT-qPCR. The 2 ^−ΔΔCT^ method was used to analyze gene expression (Jozefczuk and Adjaye, 2011).

### 2.9 Antioxidant POD enzyme activity of *N. colorata*

To evaluate the POD antioxidant enzyme activity, frozen samples (leaf, 6 h stressed) weighing 0.5 g were finely ground using liquid nitrogen and then homogenized with 4.5 ml of normal saline (0.154 M), following the protocol in the provided kit. The homogenate was subjected to centrifugation at 12,000 rpm and 4°C for 10 minutes. The resulting supernatant was transferred to a separate falcon tube for subsequent enzyme analysis. For the determination of POD activity, the instructions from kits (A084-3, Nanjing Jiancheng Bioengineering Institute in Nanjing, China) were exactly followed. The wavelength of *POD* activity was noted at 420 nm using a photometer (Lambda 25 UV/VIS Spectrophotometer).

### 2.10 Statistical analysis

Statistical analysis was accomplished using SPSS software (version 9.1, SPSS Institute Inc. Cary, NC, USA). Different stars and alphabets indicate a significant difference between the concentrations (p ≤ 0.05). For three replications of each treatment, the represented data are the means ± standard errors (SE). Fisher’s least significant difference (LSD) test was employed (p ≤ 0.05), to analyze the differences among the concentration.

## 3 Results

### 3.1 Characterization of *NcPOD* gene family in water lily (*N. colorata*)

To identify *NcPOD* family members in water lily, BLASTP and HMMER based methods were utilized to search for *NcPODs* within the water lily genome database. The 73 *Arabidopsis* POD sequences were used as queries (Tognolli et al., 2002) and the candidate sequences were confirmed through conserved domain analysis based on CDD (Conserved Domain Database). The 94 *POD* genes (*NcPOD*) were identified within the water lily genome and the genes were renamed from *NcPOD1* to *NcPOD94* (**Table 1**). The overall number of *POD* family genes in water lily was lower than that in tobacco (*N. tabacum*) (210), *S. spontaneum* (113), sorghum (150), and rice (126), but slightly higher than in *A. thaliana* (73). Similarly, the analysis of various essential information regarding the *NcPODs*, including protein identifiers, chromosomal localization, and several physicochemical properties such as protein length (aa), molecular weight (kDa), isoelectric point (pI), grand average of hydropathicity (GRAVY), and in-silico subcellular localization were conducted. The protein length of NcPODs exhibited considerable variability, ranging from 272 (NcPOD 43) to 376 (NcPOD 40) amino acids, with predicted molecular weights spanning from 29.578 to 40.784 kDa. The theoretical pI values ranged from 4.7 to 9.47, respectively. Moreover, there was diversity among most of the genes regarding GRAVY, indicating predominantly hydrophilic properties, with only a few exhibiting hydrophobic characteristics, as evidenced by positive values. Furthermore, the in-silico subcellular locations of these NcPODs were found in cytoplasm, chloroplast and mainly in extracellular.

**Table 1:** The 94 *POD* genes identified in *N. colorata* and their sequence characteristics.

Transcript ID	Gene Name	Chr.	Genomic Location	Protein Length (A.A)	MW (kDa)	pI	GRAVY	Subcellular Localization
Nycol.F00339	NcPOD1	6	3703054.3705397	320	34.115	7.55	0.134	Extracellular
Nycol.A00072	NcPOD2	1	1054123.1057093	327	34.829	7.57	0.082	Extracellular
Nycol.A02693	NcPOD3	1	34381712.34383427	322	34.982	8.32	-0.004	Extracellular
Nycol.A03280	NcPOD4	1	40216287.40218437	328	36.03	7.53	-0.065	Extracellular
Nycol.A02691	NcPOD5	1	34370060.34371707	323	35.229	6.32	-0.057	Chloroplast
Nycol.A01951	NcPOD6	1	26453240.26454914	373	40.784	4.98	-0.279	Extracellular
Nycol.A02395	NcPOD7	1	31163815.31165298	320	34.377	8.78	-0.036	Extracellular
Nycol.A02397	NcPOD8	1	31172714.31174229	320	34.112	8.79	-0.018	Chloroplast
Nycol.A02692	NcPOD9	1	34375528.34377224	322	34.782	6.09	-0.024	Extracellular
Nycol.A02728	NcPOD10	1	34692191.34694021	322	34.852	7.59	0.036	Cytoplasm
Nycol.A02398	NcPOD11	1	31177085.31178316	290	31.409	9.3	-0.248	Cytoplasm
Nycol.A02396	NcPOD12	1	31168761.31170264	320	34.017	8.4	0.02	Extracellular
Nycol.D01725	NcPOD13	4	26243253.26248297	355	40.313	5.74	-0.211	Extracellular
Nycol.D00117	NcPOD14	4	3810945.3812829	366	39.368	6.8	0.019	Extracellular
Nycol.D00118	NcPOD15	4	3856349.3858060	320	34.255	9.47	-0.072	Chloroplast
Nycol.D00127	NcPOD16	4	4064326.4093174	366	39.352	7.45	0.04	Extracellular
Nycol.B01418	NcPOD17	2	15967705.15972111	319	34.729	8.86	-0.085	Chloroplast
Nycol.B00074	NcPOD18	2	716170.721044	334	36.096	9.09	-0.157	Extracellular
Nycol.B02595	NcPOD19	2	32171393.32173582	364	40.067	8.23	-0.005	Extracellular
Nycol.B00075	NcPOD20	2	727457.729812	331	36.534	9.6	-0.298	Chloroplast
Nycol.B01417	NcPOD21	2	15960114.15963808	339	36.744	6.16	-0.035	Extracellular
Nycol.B00598	NcPOD22	2	6498528.6500246	307	33.052	4.97	-0.07	Cytoplasm
Nycol.B00079	NcPOD23	2	758330.760686	346	37.755	5.21	-0.091	Cytoplasm
Nycol.B00080	NcPOD24	2	775585.778981	337	37.722	6.46	-0.168	Extracellular
Nycol.B01420	NcPOD25	2	15976560.15980602	319	34.702	8.97	-0.109	Chloroplast
Nycol.B02559	NcPOD26	2	31747558.31749574	318	34.203	8.7	-0.055	Cytoplasm
Nycol.B01421	NcPOD27	2	15987104.15990480	318	33.557	5.07	0.14	Extracellular
Nycol.B01845	NcPOD28	2	21025135.21028223	325	35.015	7.52	0.159	Cytoplasm
Nycol.B00076	NcPOD29	2	735889.738095	327	35.562	6.5	-0.115	Extracellular
Nycol.H00999	NcPOD30	8	17528662.17530544	327	34.721	5.88	0.001	Extracellular
Nycol.H01549	NcPOD31	8	23157867.23159704	326	35.876	4.77	0.202	Extracellular
Nycol.H01452	NcPOD32	8	21928357.21930184	320	34.311	4.58	-0.051	Extracellular
Nycol.H01450	NcPOD33	8	21917982.21919804	322	35.05	5.33	-0.117	Cytoplasm
Nycol.H01407	NcPOD34	8	21509495.21512671	323	35.502	8.5	0.024	Cytoplasm
Nycol.H01449	NcPOD35	8	21912618.21914776	322	35.22	5.61	-0.135	Extracellular
Nycol.H01446	NcPOD36	8	21894753.21896529	322	34.81	5.65	-0.137	Extracellular
Nycol.H01451	NcPOD37	8	21922452.21924280	320	34.278	5.14	-0.054	Chloroplast
Nycol.J00463	NcPOD38	10	10930267.10931867	315	34.429	6.66	-0.321	Extracellular
Nycol.J00448	NcPOD39	10	10792031.10793471	341	36.731	5.91	-0.16	Chloroplast
Nycol.J00434	NcPOD40	10	10570726.10573833	376	40.611	5.91	0.114	Extracellular
Nycol.J01173	NcPOD41	10	18513166.18522803	321	34.647	6.44	-0.082	Extracellular
Nycol.J01178	NcPOD42	10	18556579.18559565	323	34.517	6.58	0.07	Extracellular
Nycol.J00450	NcPOD43	10	10808835.10810177	272	29.578	5.67	-0.272	Chloroplast
Nycol.J01175	NcPOD44	10	18534328.18536612	321	34.244	6.36	0.113	Chloroplast
Nycol.J00451	NcPOD45	10	10814959.10816412	341	36.886	6.24	-0.204	Extracellular
Nycol.J00413	NcPOD46	10	10295102.10299241	344	36.175	6.58	0.131	Extracellular
Nycol.J01176	NcPOD47	10	18540233.18544229	345	37.262	8.35	0.036	Chloroplast
Nycol.J00458	NcPOD48	10	10885480.10886829	342	37.207	5.93	-0.157	Chloroplast
Nycol.J00464	NcPOD49	10	10943742.10945580	349	37.1	5.25	-0.052	Extracellular
Nycol.J00455	NcPOD50	10	10853785.10854843	306	33.195	6.12	-0.31	Cytoplasm
Nycol.J00457	NcPOD51	10	10864872.10866272	342	37.298	5.93	-0.18	Extracellular
Nycol.J00459	NcPOD52	10	10890329.10891725	347	37.555	6.12	-0.188	Cytoplasm
Nycol.J00449	NcPOD53	10	10796565.10798053	340	36.802	6.36	-0.134	Extracellular
Nycol.J00416	NcPOD54	10	10320716.10322519	343	36.136	4.58	0.224	Extracellular
Nycol.J00456	NcPOD55	10	10858678.10860078	342	37.298	5.93	-0.18	Extracellular
Nycol.J01550	NcPOD56	10	22091770.22093383	328	35.615	8.57	-0.041	Extracellular
Nycol.J00462	NcPOD57	10	10921922.10923493	342	36.827	7.58	-0.156	Extracellular
Nycol.J00453	NcPOD58	10	10839982.10841384	337	36.724	7.19	-0.11	Extracellular
Nycol.J00417	NcPOD59	10	10328486.10330803	339	35.853	5.22	0.121	Extracellular
Nycol.J00231	NcPOD60	10	4803116.4806506	348	36.967	6.29	0.042	Extracellular
Nycol.J01222	NcPOD61	10	18961828.18964410	316	33.675	6.87	-0.022	Extracellular
Nycol.J01174	NcPOD62	10	18529070.18532262	322	34.236	4.85	0.207	Extracellular
Nycol.J00454	NcPOD63	10	10843198.10845036	368	40.111	5.67	-0.235	Extracellular
Nycol.J00435	NcPOD64	10	10576410.10579500	329	35.579	6.65	0.127	Cytoplasm
Nycol.J00460	NcPOD65	10	10911344.10912539	339	37.002	6.11	-0.138	Cytoplasm
Nycol.K01489	NcPOD66	11	19503548.19505295	326	35.27	6.94	0.011	Cytoplasm
Nycol.K01635	NcPOD67	11	20872205.20874787	326	35.312	6.11	0.091	Extracellular
Nycol.K01490	NcPOD68	11	19510053.19512152	326	35.689	6.31	-0.066	Extracellular
Nycol.M00095	NcPOD69	13	2040013.2042689	323	34.093	4.64	0.06	Chloroplast
Nycol.M00445	NcPOD70	13	7836531.7837919	321	33.92	8.08	0.058	Extracellular
Nycol.M00494	NcPOD71	13	8410578.8412252	321	33.645	7.53	0.155	Extracellular
Nycol.M00446	NcPOD72	13	7843070.7844448	321	33.927	8.08	0.058	Extracellular
Nycol.M00444	NcPOD73	13	7828812.7830364	321	33.799	8.08	0.073	Extracellular
Nycol.I01278	NcPOD74	9	18954615.18955957	324	34.91	5.02	-0.048	Extracellular
Nycol.I01196	NcPOD75	9	18017397.18020171	322	35.428	8.42	-0.065	Extracellular
Nycol.I01219	NcPOD76	9	18262528.18266668	323	35.831	6.09	-0.191	Extracellular
Nycol.I01276	NcPOD77	9	18937157.18938219	304	32.572	5.1	-0.06	Chloroplast
Nycol.I01499	NcPOD78	9	21256641.21258780	324	34.932	6.21	-0.021	Extracellular
Nycol.I01271	NcPOD79	9	18900429.18901925	324	34.336	7.56	-0.073	Chloroplast
Nycol.I01275	NcPOD80	9	18931627.18932967	324	34.826	5.2	-0.044	Extracellular
Nycol.I01273	NcPOD81	9	18913031.18914429	324	34.279	7.56	-0.059	Extracellular
Nycol.I01270	NcPOD82	9	18893453.18895414	322	34.714	5.94	-0.127	Extracellular
Nycol.C01206	NcPOD83	3	18220023.18226484	321	34.459	5.62	-0.043	Extracellular
Nycol.C01984	NcPOD84	3	25707177.25708845	320	34.365	8.95	-0.069	Extracellular
Nycol.C01205	NcPOD85	3	18206742.18209243	312	33.362	4.7	0.114	Extracellular
Nycol.C01208	NcPOD86	3	18231954.18234266	318	34.09	4.83	0.008	Cytoplasm
Nycol.C01180	NcPOD87	3	17888113.17891407	325	35.697	8.06	-0.037	Cytoplasm
Nycol.C00538	NcPOD88	3	7516154.7518788	329	36.034	7.02	0.088	Extracellular
Nycol.C01203	NcPOD89	3	18190701.18192733	320	34.229	6.31	-0.057	Extracellular
Nycol.C02174	NcPOD90	3	27537047.27539198	326	35.255	9.02	-0.091	Extracellular
Nycol.C01204	NcPOD91	3	18199586.18201568	315	34.132	5.27	-0.156	Chloroplast
Nycol.C00872	NcPOD92	3	14853754.14856515	324	35.852	8.31	-0.07	Extracellular
Nycol.C01652	NcPOD93	3	22488840.22492373	331	36.081	5.12	-0.065	Extracellular
Nycol.E01136	NcPOD94	5	19695318.19701228	326	34.857	5.4	0.028	Chloroplast

### 3.2 Analysis of conserved amino acid motifs

**Figure 1:**
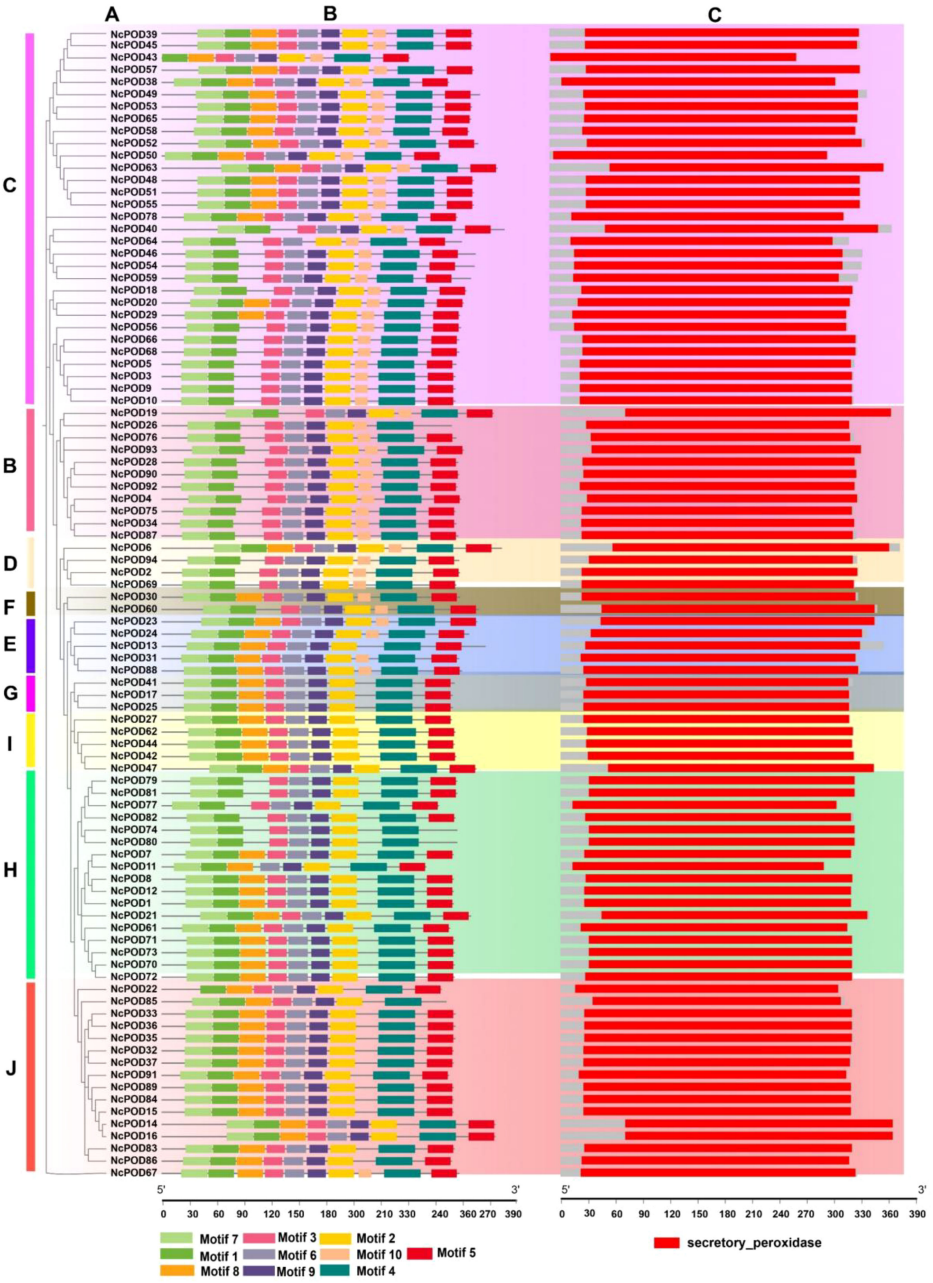
**(A)** Representation of the phylogenetic tree illustrating the relationships among NcPOD proteins **(B)** Motif composition of *POD* in the water lily, highlighted in various colors representing motifs 1–10, with non-conserved sequences indicated by grey lines. **(C)** Visualization of the conserved domains within NcPOD proteins. The presentation of motif composition and conserved domain structure was generated using TBtools software. The relative positioning is proportionally depicted on a kilobase scale at the bottom of the figure.

Molecular Evolutionary Genetics Analysis Version X (MEGA-X) was employed to make a rectangular phylogenetic tree of the 94 NcPOD proteins. The tree was constructed using the maximum likelihood method, with bootstrap value of 1000 replicates and it was organized into 10 distinct groups (**Figure 1A**). The structural characteristics of *NcPODs* were examined by identifying 10 conserved motifs through the MEME database, aligning them with the phylogenetic relationships (**Figure 1B**). The present analysis revealed that closely related *NcPODs* shared common motif compositions, suggesting potential functional similarities within the same *NcPOD* sub-group. Motifs 1–9 were highly prevalent in *NcPODs*, present in 90–98% of the proteins, while motif 10 exhibited variation across different groups, inferring at their potential significance in NcPOD proteins. The detailed information about their identified motifs, containing their names, sequences and widths is shown in S3. Additionally, an NCBI domain search and interproscan were conducted to perform a domain-based analysis of all POD proteins and used TBtools to create a comprehensive structural representation (**Figure 1C**).

### 3.3 Phylogenetic and comparative analyses of *PODs* in water lily

**Figure 2:**
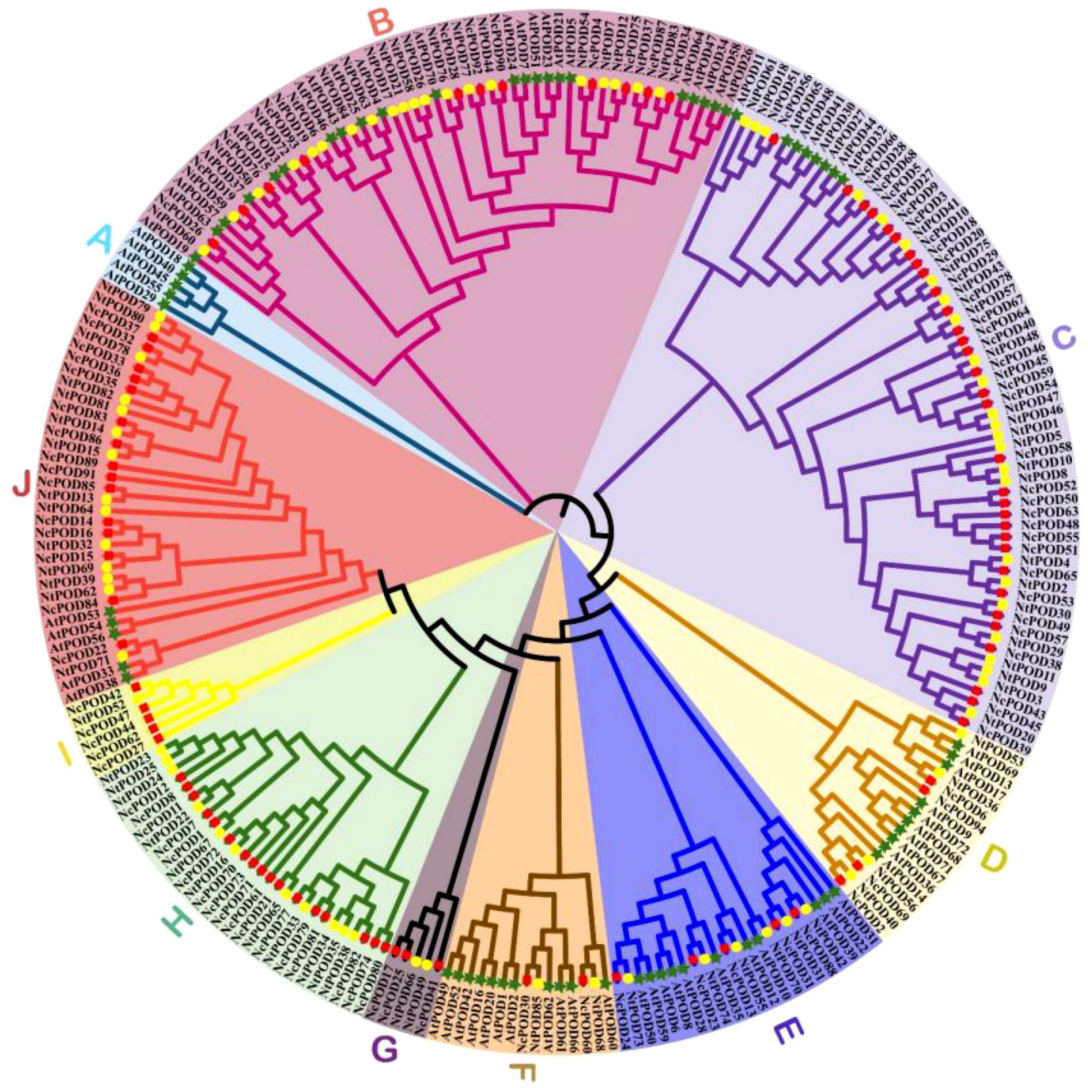
Phylogenetic relationships among *Nymphaea colorata* (red rectangle), *Arabidopsis thaliana* (green stars) and *Nymphaea thermarum POD* genes (yellow circles) based on the amino acid sequence alignment. The phylogenetic tree was generated using MEGAX with the Maximum Likelihood Method, and bootstrap support was assessed with 1000 replicates. Clusters were categorized into distinct groups (A–J) based on their evolutionary relationships.

In order to examine the evolutionary relations, 94 *POD* genes were used from *Nymphaea colorata*, 72 from *Arabidopsis thaliana*, and 85 from *Nymphaea thermarum* to create a maximum likelihood phylogenetic tree using MEGA-X. The *POD* family genes were categorized into ten distinct groups (A-J), as represented in **Figure 2**. Notably, group C was the largest, comprising 65 *POD* members, with 32 from *N. colorata*, 25 from *N. thermarum*, and 8 from *A. thaliana*. Groups B, H, and J also exhibited substantial number of genes, with 53, 28, and 34 genes, respectively. Conversely, group G was the smallest, encompassing just 5 members, including 3 of *N. colorata* and 2 of *N. thermarum*, while *A. thaliana* members were notably absent from this group. Interestingly, group A contained only members of *A. thaliana* and had no members of *N. colorata* and *N. thermarum*. Furthermore, a total of 56 orthologous pairs between *N. colorata* and *N. thermarum* were identified. However, we did not find any orthologous pairs between *N. colorata* and *A. thaliana*. This discrepancy in orthologous pairs is most likely due to the closer evolutionary relationship between the two water lily species, *N. colorata* and *N. thermarum*, as compared to the more distant relationship with *A. thaliana*.

### 3.4 Cis-acting elements in the promoter region of *NcPODs*

**Figure 3:**
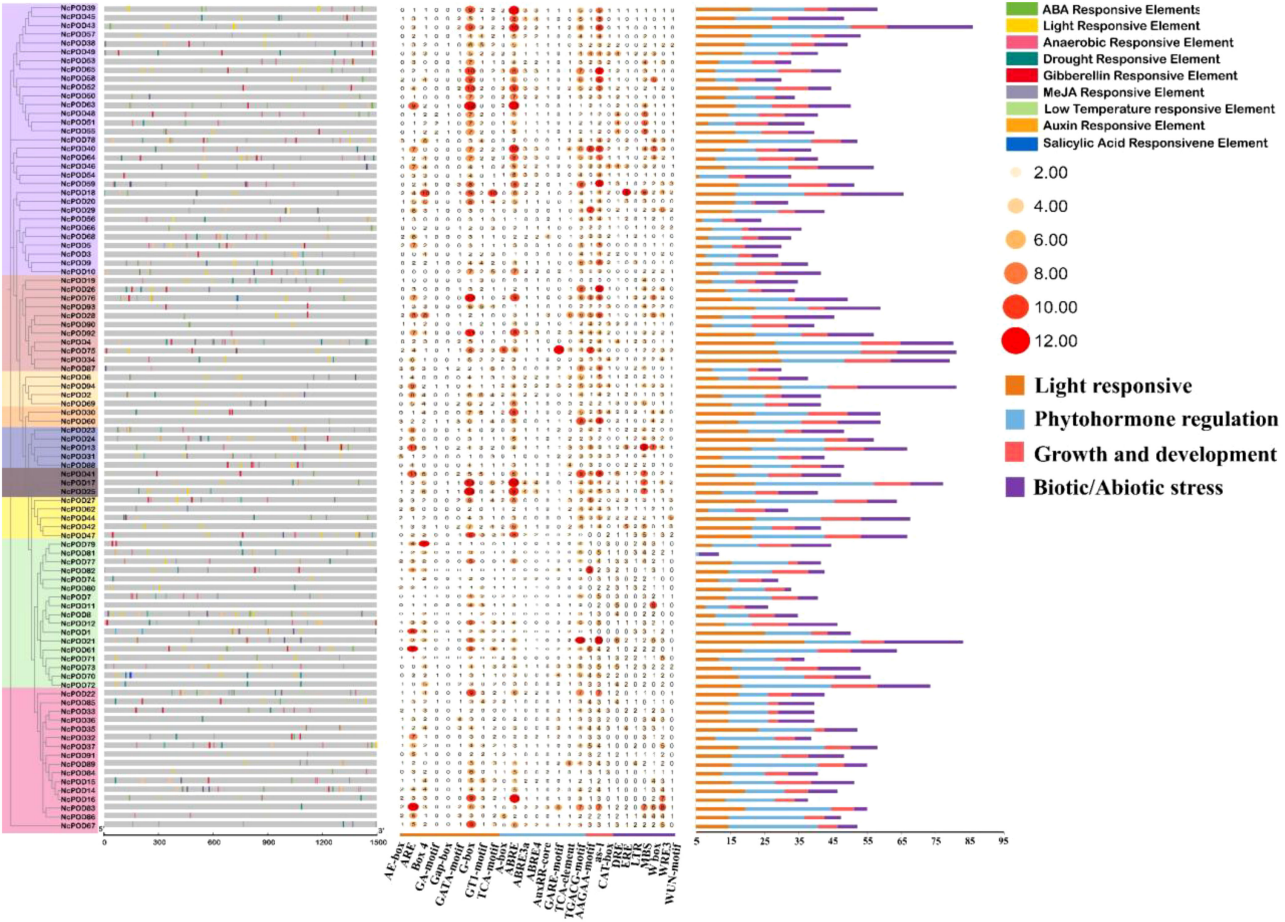
Examination of cis-regulatory elements within the 2000 bp region upstream of the transcription start site of *NcPOD* genes, with an illustration of their distribution along the promoter sequence. The cis-regulatory elements were classified based on their functional significance; including elements associated with light, stress, and hormone responsiveness. The motifs were identified using data from the PlantCARE database.

In current analysis, the cis-acting elements present in the upstream promoter region of the *NcPOD* genes were explored, which play a crucial role in regulating gene expression. The 2 kb region starting from the gene initiation site was focused on, and the PlantCARE database was used for cis-acting element analysis. The identified cis-acting elements were subsequently categorized into three distinct groups based on their putative functions: light-related, stress-related, and hormone-related elements. Notably, the largest group of regulatory elements was associated with abiotic stress, followed by elements involved in light regulation, including key regulatory motifs such as GT1-motif, G-Box, GATA-motif, and AE-Box. Additionally, hormone-related elements, encompassing CGTCA-motif, TGACG-motif, ABRE, and GARE-motif, were also prevalent (S4). This comprehensive analysis unveiled the diverse roles of *POD* gene members, highlighting their indirect contributions to various biological processes, including responses to biotic and abiotic stresses, as well as participation in hormone signalling pathways (**Figure 3**).

### 3.5 Gene structure analyses of *NcPOD* genes

**Figure 4:**
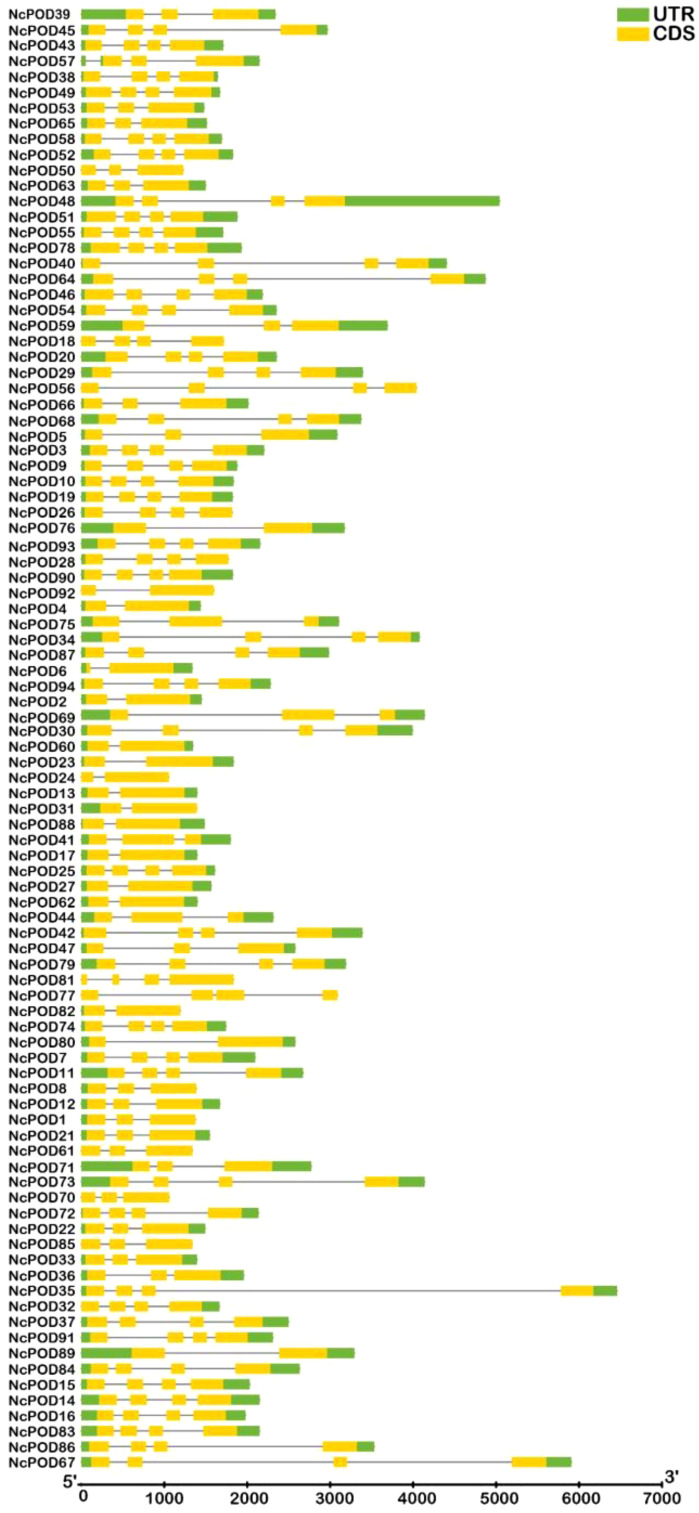
Exon/intron organization of *NcPOD* genes. Yellow boxes denote exons and black lines denote introns. The untranslated regions (UTRs) are showed by green boxes. The sizes of exons and introns can be assessed using the scale at the bottom.

This analysis provided valuable insights into the evolutionary development of structural diversity within the *POD* gene family. The exon-intron arrangement of the 94 *NcPOD* genes were examined to gain a better understanding of their structural characteristics, as illustrated in **Figure 4**. The present observations indicated that *NcPOD* genes exhibited conserved exon-intron distributions, with introns ranging from 1 to 3 and varying exon numbers between 2 and 6. While genes sharing similar exon/intron structures were grouped together, there were also occurrences of structural variation among these *NcPOD* genes. Gene structure analysis proposed that the *NcPOD* gene family generally maintained a conserved exon/intron organization.

### 3.6 Chromosomal locations and dual synteny analysis of *NcPOD* genes

**Figure 5:**
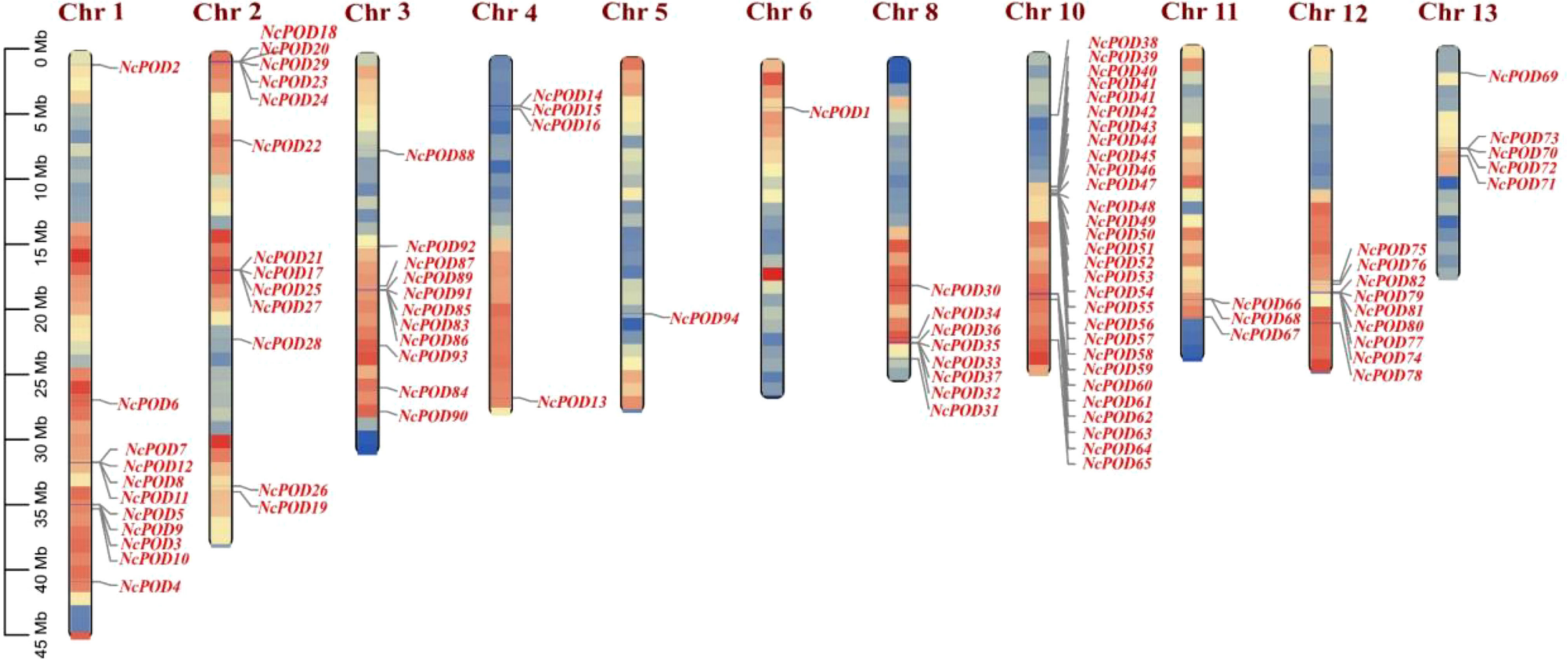
Genomic localization and distribution patterns of *NcPOD* genes in the *N. colorata* genome. The scale on the left presents the length of *N. colorata* chromosomes (Mb). The colors on the chromosomes denote gene density.

To elucidate the genome organization and chromosome distribution of *NcPOD* genes in the water lily, a chromosomal map was generated using TBtools. In this map, a total of 94 *NcPOD* genes on the *N. colorata* chromosomes were identified (**Figure 5**). Notably, there was not found the distribution of any *POD* gene on chromosomes 7, 9 and 14. The distribution of *NcPOD* genes across the 11 chromosomes was uneven. Amongst them, the highest number of *NcPOD* genes (29) was located on chromosome 10, followed by chromosomes 2 (13), and chromosomes 1 and 3 had same number of genes (11). Conversely, only a few *NcPOD* genes were found on chromosomes 4 (4), 8 (8), 11 (3), 12 (9) and 13 (5), while the chromosome 5 and 6 contained only single gene. Additionally, some chromosomes showed dense clusters of *NcPOD* genes, particularly near the telomeric regions of chromosomes 2, 8, and 12. By mapping these patterns of uneven distribution and gene clustering, the chromosomal positioning was due to the evolutionary history of this species. The existence of clustered *NcPOD* genes suggests regions of the genome that may have experienced tandem duplication events, potentially contributing to the rapid diversification and functional expansion of this gene family.

**Figure 6:**
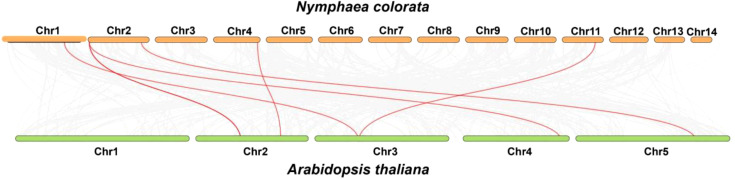
Genome-scale dual synteny plot between *N. colorata* and *A. thaliana* genomes, with syntenic *POD* orthologous genes shown in red connecting lines. Grey lines highlight the collinear blocks between the *N. colorata* and *A. thaliana* genomes.

A dual synteny plot-based in-depth comparative analysis of *PODs* between *N. colorata* and *A. thaliana* genomes was conducted. In summary, the genes presented on Chr01, Chr02, Chr04, and Chr11, demonstrated syntenic relations with *A. thaliana*, respectively. In contrast, Chr03, Ch05, Ch06, Ch07, Ch08, Ch09, Ch10, Ch12, Ch13, and Ch14, did not show any syntenic relations with *A. thaliana* (**Figure 6**). The results indicated that water lilies and *Arabidopsis* do not have a close relationship.

### 3.7 Expression analysis of *NcPOD* genes under various abiotic stresses

Antioxidant enzymes play a vital role in scavenging reactive oxygen species and defending cells against oxidative stress from both biotic and abiotic stressors (Li et al., 2018). Peroxidase (*POD*) enzymes are a vital group of antioxidant enzymes that play a crucial role in protecting cells from oxidative damage caused by reactive free radicals (González-Gordo et al., 2023). In this context, the expression patterns and responses of *POD* antioxidant genes in *N. colorata* under various abiotic stress conditions were investigated using RT-qPCR analysis.

**Figure 7:**
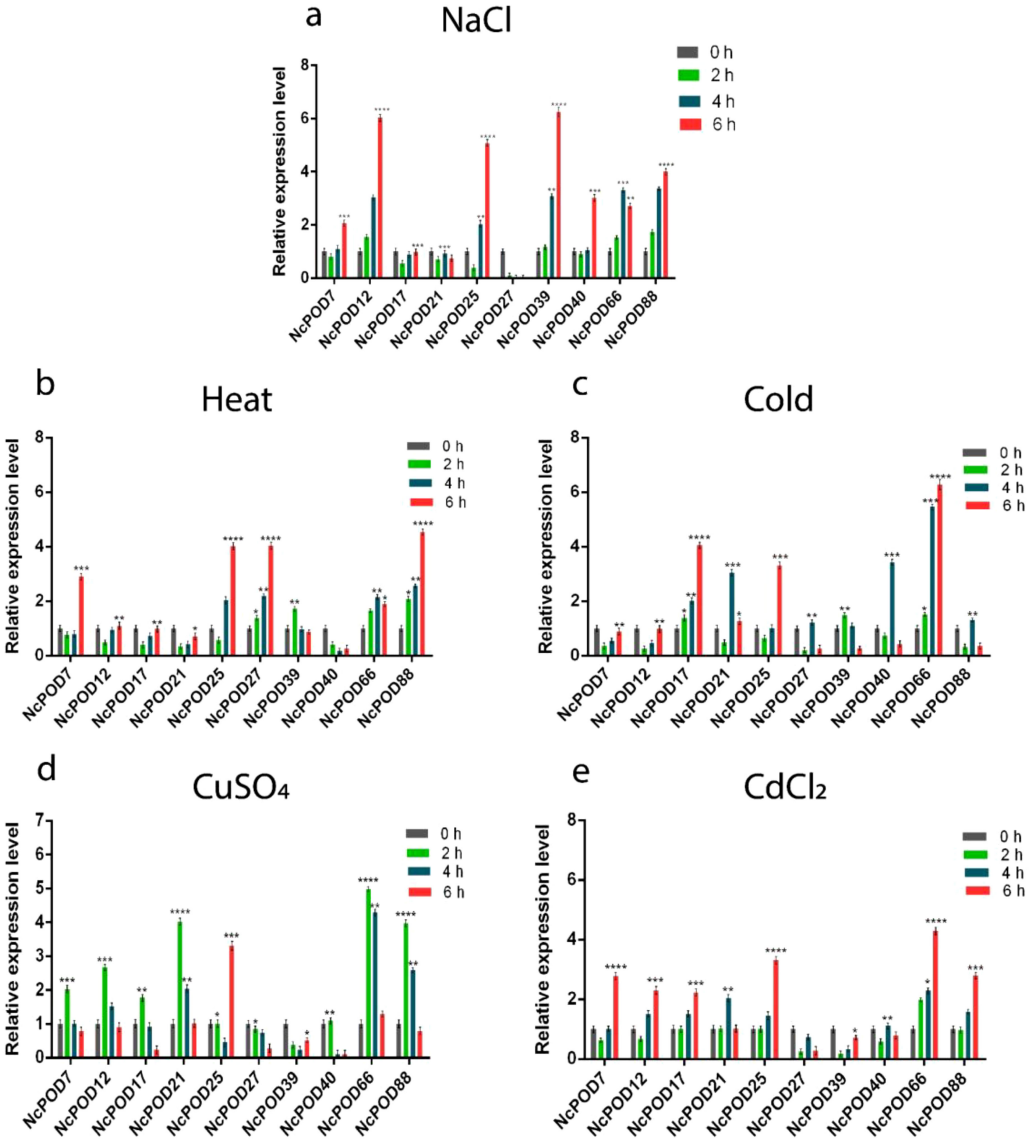
The expression pattern of *POD* genes in water lily leaves under various abiotic stresses. *POD* genes expression in *N. colorata* under **(A)** NaCl; **(B)** heat; **(C)** cold; **(D)** CuSO_4_; and **(E)** CdCl_2_ stress at different time intervals (0 (CK), 2, 4, and 6 h). Data presented as means, ± standard error, n = 3; statistically significant differences are exhibited by asterisks (p ≤ 0.05). The (*) symbols indicated statistically significant differences between treatments. Following the values of each star. (*p* < 0.05 => *, *p* < 0.01 => **, *p* < 0.001 =>***, *p* < 0.0001 =>****).

During salinity stress, the *NcPODs* showed variable expressions. Among them the *NcPOD7, NcPOD12*, *NcPOD25*, *NcPOD39, NcPOD40* and *NcPOD88* exhibited initially decreased expression and then reached their highest peak at 6 h. Similarly, a single *NcPOD27* gene was found downregulated (**Figure 7A**).

In response to heat stress, approximately 55% of the total genes exhibited elevated expression levels, while the remaining genes displayed either moderate or low expression. The *NcPOD7*, *NcPOD25*, and *NcPOD88* showed a significant upregulation over time, with peak expression at 6 h. *NcPOD39* exhibited higher expression at 2 h, which then decreased at 4 and 6 h. A single *NcPOD66* gene was upregulated at 4 h (**Figure 7B**).

When the *NcPOD* genes were exposed to low temperature, fluctuations in expression were observed among the genes, with significant expression recorded at 6 h. Like the *NcPOD17*, *NcPOD25*, and *NcPOD66* exhibited temporal upregulation expression. Additionally, *NcPOD21*, *NcPOD40*, and *NcPOD88* were upregulated and exhibited a higher expression at 4 h (**Figure 7C**).

During heavy metals treatment, the *POD* genes exhibited dynamic expression. In response to CuSO_4_ treatment, the *NcPOD* genes showed differential expression level. Among the 10 genes studied, *NcPOD7*, *NcPOD12*, *NcPOD17*, *NcPOD21*, *NcPOD66*, and *NcPOD88* exhibited high expression levels at 2 h, followed by a decrease in expression by 6 h. However, a single gene *NcPOD25* revealed the highest expression at 6 h (**Figure 7D**).

Under CdCl_2_ treatment the *NcPOD7*, *NcPOD12*, *NcPOD17*, *NcPOD25*, *NcPOD66*, and *NcPOD88* showed an upregulated temporal expression. However, the *NcPOD21* and *NcPOD40* exhibited a higher expression at 4 h (**Figure 7E**).

### 3.8 Enzymatic activity of *NcPOD* genes of *N. colorata*

**Figure 8:**
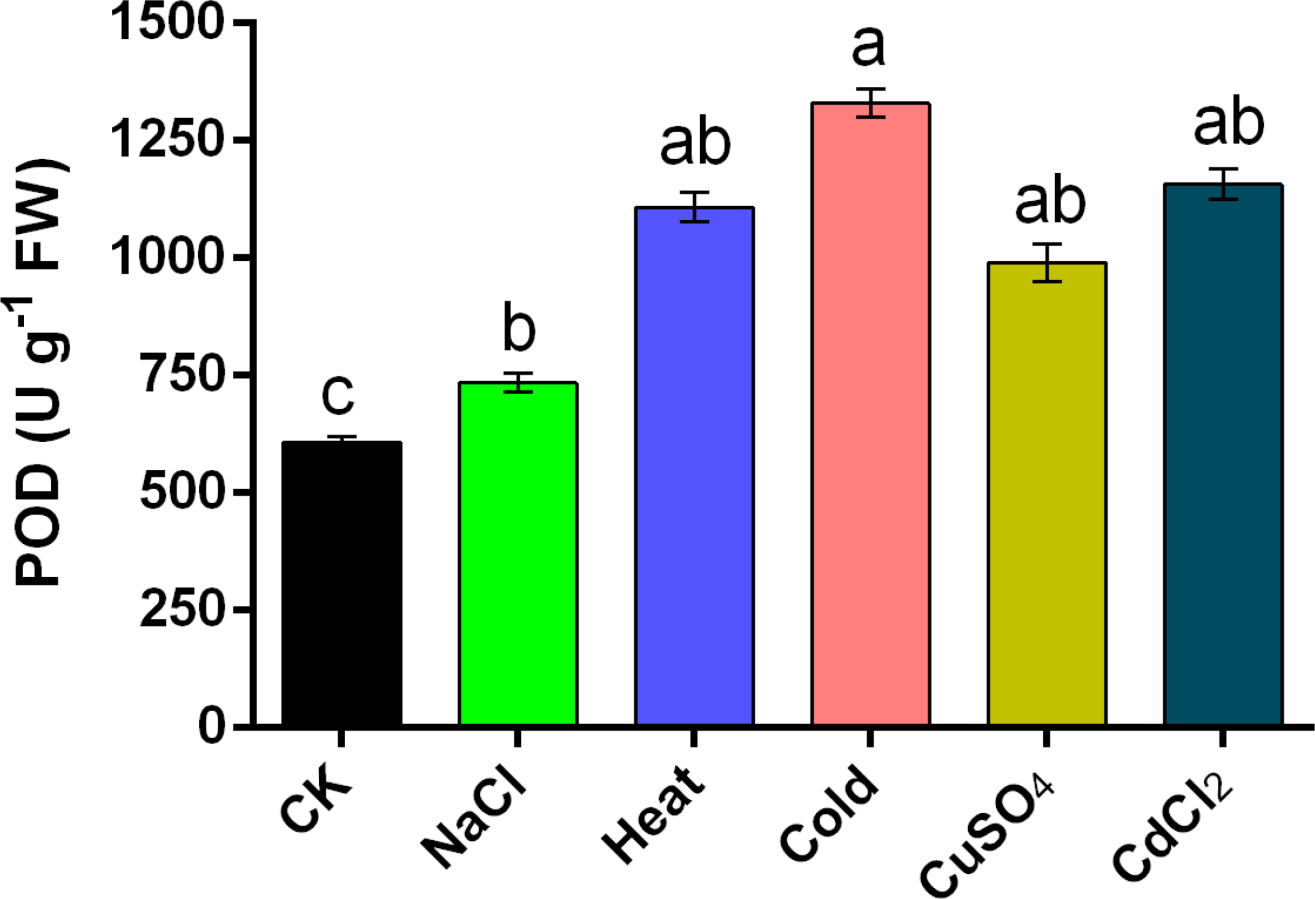
Concentration of POD enzymes under multiple abiotic stresses, such as salinity, heat, cold, CuSO_4_, and CdCl_2_ in Nymphaea colorata. The standard error of the means (n = 6) represented by the bars. Different lower-case letters represent significant differences among treatments at P ≤ 0.05, according to LSD test.

In addition to the gene expression measurements, analyses of the 6 h stressed enzyme activity of *NcPOD* genes of *N. colorata* plant subjected to various abiotic stresses were conducted. The total enzyme content of POD was assessed. In particular, the comprehensive POD activity of *N. colorata* displayed a pattern of enhancement, demonstrating an accumulation of 200.8%, 242.16%, 278.4%, 74.4%, and 139.6% in the aforementioned stresses as compared to control (**Figure 8**). These results were found almost consistent with the RT-qPCR based expression analysis of *POD* genes suggesting that stress response was under genetic control in water lily specie.

## 4 Discussion

Plants grown in natural environments constantly face various survival stress. Abiotic stress usually refers to adversity growth conditions like drought, salinity, extreme heat, cold, and ultraviolet (UV) which can affect plant growth (Han et al., 2023). Class III Peroxidases are known to be involved in various physiological processes within plants (Tognolli et al., 2002; Ren et al., 2014) and perform a crucial role in both biological and abiotic stress responses during plant development (Wang et al., 2015). Therefore, it is essential to systematically and comprehensively characterize the *POD* gene family at the genome scale in plant species to understand their contributions to plant growth and defense responses. Although *POD* gene families have been studied in *A. thaliana* (Tognolli et al., 2002), *Populus trichocarpa* (Ren et al., 2014), *Zea mays* (Wang et al., 2015), and *Oryza sativa* (Passardi et al., 2004a), there is a lack of information regarding the identification and function of the *POD* gene family in water lilies. Fortunately, with the completion of the full genome sequence of *N. colorata*, it has become possible to conduct bioinformatics analyses of the *POD* gene family at the genome level (Zhang et al., 2020). This study aimed to systematically characterize the *POD* family on a genome-wide scale in *N. colorata* and verify its role in abiotic stress responses. The analysis revealed the identification of 94 *PODs* in the *N. colorata* genome through a comprehensive genome-wide approach. This number surpasses those reported in *Arabidopsis thaliana* (73), cassava (91), and *P. trichocarpa* (93) (Lüthje et al., 2011), but is lower compared to tobacco *N. tabacum* (210) and *O. sativa* (138) (Passardi et al., 2004a). This suggests a significant expansion of the *POD* gene family in *N. colorata*, *N. tabacum* and *O. sativa* compared to other species. The study encompassed a thorough analysis of physicochemical properties, phylogenetic relationships, gene synteny, motif composition, gene structure organization, and cis-regulatory elements. Additionally, RT-qPCR analysis and enzyme concentrations in response to abiotic stresses (NaCl, heat, cold, and heavy metals) provided extensive information on gene functions and expression dynamics related to applied stress responses in water lily.

Comparative phylogenetic examination of *N. colorata*, *N. thermarum*, and *A. thaliana POD* family genes were divided into ten distinct groups (A-J). The analysis revealed a robust clustering relationship with closely related species, indicating a conserved evolution of this gene family. This suggests that this gene family has experienced relatively conserved evolution, almost consistent with findings on cassava (Wu et al., 2019).

In current analysis, ten highly conserved motifs present in the 94 NcPOD proteins were identified. Notably, there were slight variations in both the number and type of these conserved motifs among the proteins. Interestingly, the majority of NcPOD proteins contained all these conserved motifs, suggesting their potential involvement in the fundamental functions of POD proteins. The diversity in gene structure is known to play a significant role in the evolution of gene families (Muthamilarasan et al., 2014; Han et al., 2016). This study examined into the structure of *NcPOD* genes, revealing variations in the number of exons and introns. It is noteworthy that stress-related genes typically exhibit fewer introns (Jeffares et al., 2008). Consistent with this observation, *NcPOD* genes were found to have a maximum of three or fewer than three introns, confirming this conceptual framework. This is consistent with previous reports on *POD* genes in various plant species, such as maize (Wang et al., 2015), cassava (Wu et al., 2019), wheat (Yan et al., 2019), and Chinese pear (Cao et al., 2016), which also showed a lower number of introns. The similarities in gene structure and motif composition within each *NcPOD* subgroup support the phylogenetic classification proposed in this study.

As part of the characterization of the *NcPOD* genes, the presence of cis-regulatory elements in 2000 bp upstream regions from the transcription starting point of the 94 *NcPOD* genes was evaluated. The identified cis-acting elements were then classified into three different groups based on their potential functions i.e related to light, stress, and hormones. Particularly, the predominant group of regulatory elements was linked to abiotic stress, followed by elements associated with light regulation, which included significant regulatory motifs such as GT1-motif, G-Box, GATA-motif, and AE-Box. These cis-elements are likely contributors to the regulation of gene expression under various stress conditions. Similar findings have been reported in diverse plant species, such as Pepper (*Capsicum annuum L*.) (González-Gordo et al., 2023), sugarcane (Shang et al., 2023), cassava (Wu et al., 2019) and tobacco (Cheng et al., 2022).

Gene family expansion usually occurs through three main mechanisms: segmental, tandem and whole-genome duplication (Freeling, 2009; Cao and Shi, 2012). To explore the duplication modes of *POD* genes in the water lily, the chromosomal locations of the *NcPOD* genes were determined. Chromosomal mapping displayed that these genes are spread across 11 of the 14 chromosomes in *N. colorata* (**Figure 5**). Accumulated evidence has shown that duplication events play a crucial role in the expansion of genes within the *POD* family. This wide distribution is consistent with the chromosomal distribution of *POD* genes observed in other plants such as *Arabidopsis*, rice, *Populus trichocarpa*, maize and *Pyrus bretschneideri* (Tognolli et al., 2002; Passardi et al., 2004a; Ren et al., 2014; Wang et al., 2015; Cao et al., 2016).

Numerous researchers have underscored the involvement of plant peroxidases in a various of cellular processes throughout plant growth and development, and its consequences to abiotic and biotic stress have been reported over the years (Xue et al., 2008; Wang et al., 2015). Notably, *NcPOD* genes demonstrated significant variations in their expression profiles, highlighting their roles in various stress and defense responses in *N. colorata.* Meanwhile, individual *POD* genes typically exhibited sensitivity to specific external stresses, with few genes responding broadly to a variety of biotic and abiotic stresses. For instance, all selected *NcPOD* genes revealed responsiveness to numerous applied stresses, including heat, cold, salt, and heavy metals. Under salinity stress, among the genes of *N. colorata* a single gene (*NcPOD88*) exhibited the highest expression. These findings are consonant with earlier studies that highlighted differential expression patterns in grapevine (*Vitis vinifera L.)* under conditions of salinity stress (Xiao et al., 2020). The results unveiled diverse responses across all genes under heat stress, exhibiting varying levels of high, moderate or low expression in comparison with control. The genes i.e (*NcPOD7, NcPOD12, NcPOD17, NcPOD25, NcPOD27*, and *NcPOD88*) also exhibited significant expression. Comparable outcomes were observed in the context of heat stress treatment in potato (*Solanum tuberosum L.*), further affirming the consistency of these findings (Yang et al., 2020). Upon exposure to cold treatment, the *NcPOD* genes revealed upregulation expression pattern. Similar findings were observed in Cassava, under cold treatment (Wu et al., 2019). During heavy metal stress, the *N. colorata* exhibited variable expressions. Specifically, during CuSO_4_ treatment, a single gene (*NcPOD25*) exhibited a highest expression at 6 h. Under CdCl_2_ treatment as shown in the figure (**Figure 7E**), the *PODs* were significantly expressed and reached their expression at 6 h respectively. In this study, the expression of genes were in agreement with those detected in white clover (*Trifolium repens*) under Cd treatment (Zhang et al., 2015). The enzymatic activity of *PODs* in *N. colorata* was found to be almost consistent with the RT-qPCR-based expression analysis of antioxidant genes, indicating that the stress response in this water lily specie is under genetic control.

## 5 Conclusion

In this study, a genome-wide examination and comprehensive analysis of the *POD* gene family in water lily was conducted. In conclusion, a total of 94 *POD* genes were systematically identified in water lily (*N. colorata*) and categorized into 10 sub-groups, as supported by phylogenetic analysis. The structural diversity of *NcPODs* may reflect their functional diversity. The analysis of expression patterns of *NcPOD* genes and enzyme accumulation showed that these genes were expressed distinctly, and some might be linked to stress responses. Consequently, the present findings contribute significantly to the understanding of *POD* genes in water lilies, serving as a robust foundation for genetic enhancement in other aquatic plants. Future investigations will focus on gene engineering and comprehensive analysis, incorporating genomics, transcriptomics, proteomics, and metabolomics to further elucidate the functions of *NcPODs.*

## Data Availability

The original contributions presented in the study are included in the article/**Supplementary Material**. Further inquiries can be directed to the corresponding author/s.
