# Supplementary figures and images for: Comprehensive identification, characterization and expression analyses of the class III POD gene family in water lily (Nymphaea colorata)
Source: Front Plant Sci. 2025 Jan 20;15:1524657. doi: 10.3389/fpls.2024.1524657 (PMC11788295; doi:10.3389/fpls.2024.1524657)

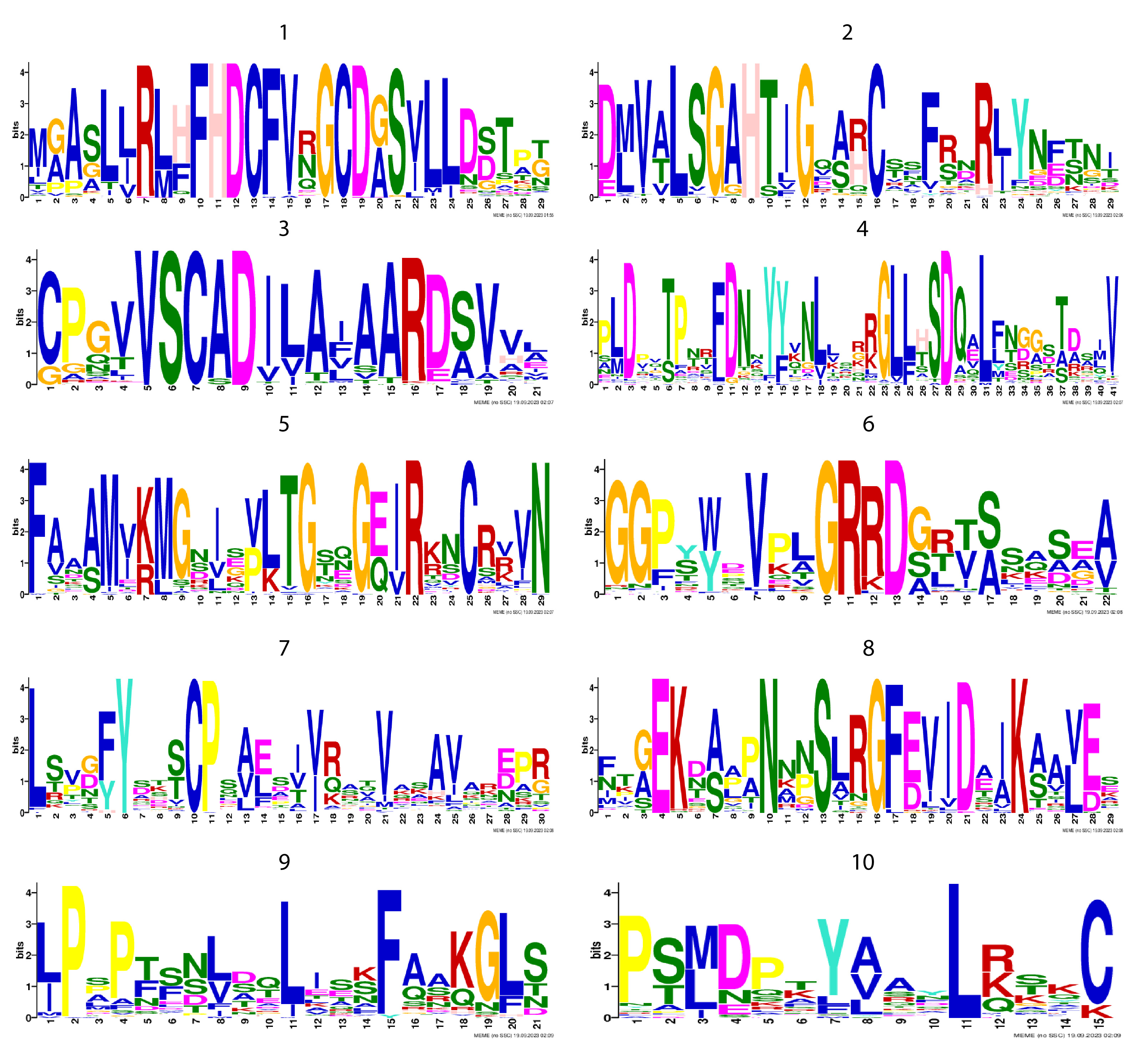

Supplement: Supplementary file 1 [file DataSheet1.zip › Motif logos.jpg]
